# A Fortunate Hospital

**Published:** 1890-07-12

**Authors:** 


### Passing Topics.

### A FORTUNATE HOSPITAL.

"The Earl of Leicester, who has come back from the Continent in much improved health, has offered to endow the new Norfolk and Suffolk Hospital with the sum of £20,000, so as to secure the financial independence of the Institution. Mr. Fletcher, a wealthy citizen, has agreed to build the hospital as a family memorial." Such is the paragraph which appeared in a daily contemporary a few mornings ago, and it is perhaps scarcely to be wondered at that the member of the editoral staff responsible for its making up, in his hurry to give so auspicious an announcement to the world, should have placed it somewhat incontinently in the column devoted to "London, day by day," or that the writer should have substituted an imaginary charity for the well-known Norfolk and Norwich Hospital, to which the addition is to be made.

Or suppose the announcement was put where it appeared by cunning design, in order to strike the eye and appeal to the senses of the reader the more readily, because of the incongruity of its position. It is not the hospitals of London which can boast of friends to endow them nowadays. Such munificence is essentially provincial, but it deserves, as it obtains, the honours of a metropolitan notice, and, in this instance, as if to point a moral, the record figures in proximity to an intimation that a certain gentleman has expended a somewhat similar sum upon the purchase of three pictures.

It is said that the publicity accorded to great crimes induces efforts to imitate them, and pity it is if deeds of philanthropy and virtue exercise no such spell upon the imagination of those who have the power of reproduction. For many a year the metropolitan hospitals have hoped against hope, and longed for that good time which a certain inspiriting ditty always assures us is coming, but which in their case seem so slow to appear. Not a few friends were sanguine that the Jubilee year would prove a veritable argosy, laden with treasure, and they might well be pardoned if their thoughts lingered wistfully about the idea, and that they dreamed pleasantly of a short period of plenty for the hospitals, though it proved no more than enough to renew their strength and vigour against the inevitable recurrence of famine. Alas ! that the propitious period passed away barren of all but disappointment, and when posterity asks what was done during the Jubilee year for institutions devoted to the relief of sickness and suffering the record will be silent. Strange it is that while riches multiply and opportunity increases, the present generation of Englishmen cannot compare with some that have gone before in notable acts of personal philanthropy. In the hospital world the collection of £100,000 for Guy's supplies the chief example of great things accomplished, but it must be remembered that this fund took a considerable time to raise, and represented the offerings of a vast number of persons, while, as if to point our contention, the chief contributor was an American citizen, who showed our countrymen, and not in this matter only, where their duty lay.

The present time is distinguished for its criticism—we had almost written cynicism—in respect of things charitable, and hospitals whose value had been almost supposed self-evident, are called upon to justify their very being. Long enthroned in the hearts of the benevolent, they are now exposed to the fulness of that fierce light which the Laureate assures us beats upon a throne. The Norfolk and Norwich Hospital is amongst the best of the provincial institutions, and well deserves its friends. Metropolitan hospitals will not envy its good fortune, but many of them will wish they too could boast supporters gifted not only with generosity but with a rare aptitude for applying it judiciously—that is, in portions large enough to be lasting. Agricola.

